# Pseudomyxome péritonéale résultant d'un tératome ovarien associé à une tumeur mucineuse bordeline: à propos d'un cas et revue de la littérature

**DOI:** 10.11604/pamj.2013.14.156.2589

**Published:** 2013-04-24

**Authors:** Amina Mohtaram, Saber Boutayeb, Meryam Ben Ameur El Youbi, Tanae Sghiri, Imane Aaribi, Fouad Kettani, Hind M'rabti, Hassan Errihani

**Affiliations:** 1 Service d'Oncologie Médicale, Institut National d'Oncologie, Rabat, Maroc; 2 Centre d'Anatomie Pathologique Nations Unies, Rabat, Maroc

**Keywords:** Pseudomyxome péritonéal, tumeur mucineuse ovarienne, tératome ovarien, traitement, pronostic, pseudomyxoma peritonei, mucinous ovarian tumor, ovarian teratoma, treatment, prognosis

## Abstract

Le pseudomyxome péritonéal est défini par la présence de mucine extra-cellulaire dans la cavité péritonéale. Il est dû dans la majorité des cas à la rupture intrapéritonéale d'une tumeur mucineuse d'origine appendiculaire avec extension secondaire ovarienne. Nous rapportons le cas d'une patiente opérée pour tumeur ovarienne droite et dont l’étude histologique était en faveur d'un pseudomyxome péritonéal sur tératome ovarien associé à une tumeur mucineuse bordeline.

## Introduction

Le pseudomyxome péritonéal ou la maladie gélatineuse du péritoine est une pathologie rare. Son incidence est estimée de 1 à 2 cas par an et par million d'habitant avec une prédominance féminine [1]. L'origine du pseudomyxome péritonéal reste sujette à controverse. Grâce à l'immunohistochimie et au génie moléculaire, il est admis que l'origine est appendiculaire dans environ 90% des cas [2, 3]. Cependant les tératomes kystiques matures associés aux tumeurs mucineuses ovariennes bordelines représentent une exception à cette règle [4]. A travers ce cas clinique nous soulevons la difficulté du diagnostic et la prise en charge thérapeutique.

## Patient et observation

Il s'agissait d'une patiente âgée de 43 ans sans antécédents pathologiques particuliers et qui présentait depuis un an une aménorrhée associée à des douleurs pelviennes.

A l'examen clinique on notait la présence d'une masse abdomino-pelvienne sensible. L’échographie pelvienne révélait la présence en rétro-utérin d'une masse anéchogène à paroi fine et double composante tissulaire et liquidienne associées à des végétations endokystiques. Découverte en per-opératoire d'une énorme masse gélatineuse pesant un kilogramme avec présence d'une tumeur ovarienne droite sans végétations exokystiques. Une chirurgie de cyto-réduction a été réalisée consistant en l'extraction de la totalité de la gélatine avec hystérectomie totale, annexectomie bilatérale, appendicectomie et ommentectomie.

**Figure 1:**
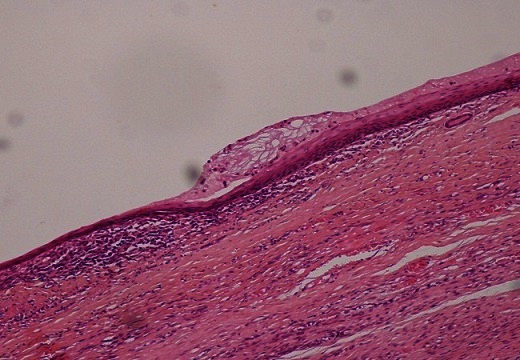
Aspect microscopique (grossissement x100) montrant un épithélium de revêtement malpighien

**Figure 2:**
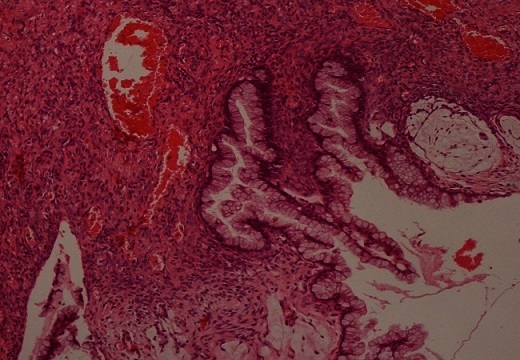
Présence d'une composante cylindrique muco-sécrétante

**Figure 3:**
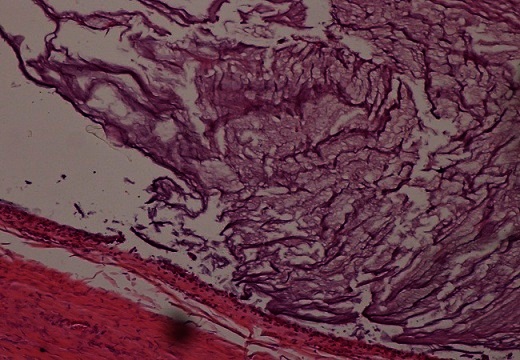
Présence de contenu mucoide acellulaire

**Figure 4:**
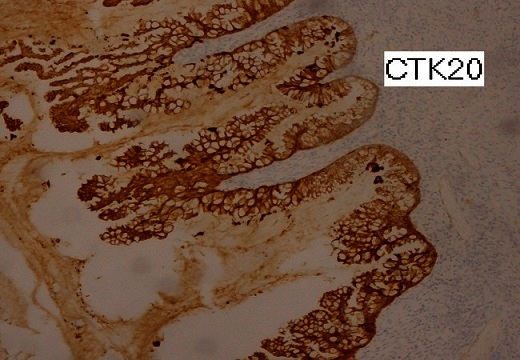
En immunohistochimie on note la positivité d'expression du CK20

Macroscopiquement, l'ovaire droit présentait à sa surface une brèche capsulaire au niveau de laquelle s’écoulait un matériel mucineux. On notait à la coupe un aspect kystique à contenu mucoide accompagné d'un tissu graisseux et de nombreux cheveux. Sur le plan microscopique, l'annexe droite comportait une formation kystique de nature tératomateuse caractérisée par un épithélium de revêtement de type malpighien subissant une maturation cornée (Figure 1). On notait aussi la présence d'une composante cylindrique muco-sécrétante uni ou pseudostratifié avec desquamation en touffes sans infiltration de la paroi (Figure 2). La pièce d'appendicectomie montrait des lésions d'endoappendicite chronique oblitérante sans composante tumorale visible. L'annexe gauche ainsi que la pièce d'ommentectomie étaient indemne de toute prolifération tumorale, avec présence d'un contenu acellulaire (Figure 3). A L’étude immunohistochimique, les cellules exprimaient le CK20 avec négativité au CK7 (Figure 4). L'aspect était en faveur d'un pseudomyxome péritonéal développé à partir d'un tératome ovarien mature avec tumeur mucineuse Borderline.

La tomodensitométrie thoraco-abdomino-pelvienne post opératoire était normale ainsi que les marqueurs tumoraux, notamment le CA125 à 15 UI/ml et le CA19-9 < 2 UI/ml. La patiente a été mise sous surveillance, avec absence de signe de récidive intrapéritonéale après un suivi de 6 mois.

## Discussion

Le pseudomyxome péritonéal est une entité clinico-pathologique rare, décrite pour la première fois par R. Wyerth en 1884. Il décrivait l'accumulation de mucine extracellulaire dans la cavité péritonéale avec présence d'une tumeur mucineuse ovarienne [5]. Puis en 1901, Frankel l'associe à la présence d'un mucocèle appendiculaire [6]. L'origine du pseudomyxome péritonéal chez la femme est restée pendant longtemps un sujet à controverse. Il a été démontré grâce aux études immunohistochimiques portant sur les antigènes CK7, CK20, et HAM-56, que l'origine du pseudomyxome était le plus souvent appendiculaire et non ovarienne [7]. Ceci a été renforcé récemment par les données de la biologie moléculaire concluant que la sur-expression du gène MUC-2 dans le pseudomyxome serait la conséquence de la présence de bactéries gram-négatives (issues de la perforation de l'appendice). Cette surexpression est corrélée à la densité des germes et à un mauvais pronostic [2]. Entre outre, l′étude des mutations du gène K-ras et la perte d′allèles au niveau des chromosomes 18q, 17p, 5q et 6q sont constatées dans le pseudomyxome péritonéal et ne sont pas trouvées dans les vraies tumeurs borderline de l'ovaire [3].

Les tumeurs mucineuses ovariennes, considérées comme étant à l'origine des pseudomyxomes, peuvent donner des implants tumoraux péritonéaux appelés carcinose péritonéale, mais pas de pseudomyxome véritable. Les seules tumeurs ovariennes primitives capables d'une authentique dissémination pseudomyxomateuse seraient les tératomes kystiques matures, liée probablement à l'existence d'un contingent gastro-intestinal dans ces tumeurs embryonnaires [4].

L′association de pseudomyxome péritonéal avec tératomes kystiques matures ovariens est retrouvée dans 3 à 8% des cas [8]. Deux études récentes dont une portant sur des tumeurs mucineuses de l′ovaire de type intestinal, a rapporté 3 cas de pseudomyxome péritonéal associé au kyste dermoide de l'ovaire [9]. Deux cas avaient un appendice normal, sans signe de récidive après 5 et 16 ans. La deuxième étude a décrit trois cas de tumeurs mucineuses de l′ovaire avec tératomes kystique [10]; l′appendice était normal et les tumeurs mucineuses présentaient un marquage positif du CK20 et un marquage négatif du CK7. Les lésions péritonéales étaient en faveur d'adénomucinose péritonéale disséminée dans deux cas et intermédiaire dans le 3ème. Pas de données sur le suivi.

En général les tumeurs mucineuses ovariennes sont généralement CK7 positifs avec une expression variable de CK20, contrairement à ceux associés aux tératomes matures, qui sont CK20 + / CK7- [10].

Le traitement du pseudomyxome péritonéal n'est pas encore standardisé et aucune donnée de la littérature ne permet d′établir des conclusions claires. Néanmoins l'exérèse complète par chirurgie de cytoréduction suivie de chimiothérapie hyperthermique intrapéritonéale donne de bons résultats en termes de survie, et reste à ce titre considérée comme le « gold standard » thérapeutique [8].

Aucune récidive intrapéritonéale n'a été rapportée pour le pseudomyxome péritonéal d'origine ovarienne [9]. La chimiothérapie adjuvante par 6 cycles de paclitaxel et carboplatine a été décrite dans des cas cliniques [4, 8]; mais aucune étude n'a démontré le bénéfice de cette chimiothérapie [4].

Ainsi notre observation est un cas rare de pseudomyxome péritonéal résultant d'une tumeur ovarienne et soulevant des points essentiels: L′appendice était normal sur le plan histologique, suggérant une tumeur mucineuse ovarienne primitive et non secondaire. La positivité d'expression du CK20 avec négativité d'expression du CK7 suggérait une origine gastro-intestinale mais l'appendice était normal. Enfin la présence d′un épithélium malpighien avec des glandes sébacées indiquait la présence d′un tératome mature kystique.

## Conclusion

Le pseudomyxome péritonéal est certes d'origine appendiculaire dans la majorité des cas mais son origine ovarienne est probable quand l'appendice est normal. Ceci est d'importance lorsque l'on prend conscience que le pronostic des pseudomyxomes appendiculaires est bien plus sombre que celui des tumeurs mucineuses “borderline”; de l'ovaire [4]. Le traitement curatif reste la cytoréduction suivie de chimiothérapie hyperthermique intrapéritonéale. L'intérêt de la chimiothérapie adjuvante reste incertain.

## References

[1] Smeenk RM, Van Velthuysen ML, Verwaal V, Zoetmulder F (2008). Appendiceal neoplasms and pseudomyxoma peritonei: a population based study. EJSO..

[2] O'Connell JT, Tomlinson JS, Roberts AA (2002). Pseudomyxoma peritonei is a disease of MUC2-expressing goblet cells. Am J Pathol..

[3] Szych C, Staebler A, Connolly DC (1999). Molecular genetic evidence supporting the clonality and appendiceal origin of pseudomyxoma peritonei in women. Am J Pathol..

[4] Manmeet Saluja, Diane N, Kenwright, John P Keating (2010). Pseudomyxoma Peritonei arising from a mucinous borderline ovarian tumour: Case report and literature review. Aust N Z J Obstet Gynaecol.

[5] Werth R (1884). Klinische und anatomische untersuchungen zur lehre von den bauchgeschwuelsten und der laparotomie. Arch Gynecol Obstet..

[6] Frankel E (1901). Uber das sogenannte pseudomyxoma peritonei. Med Wochenschr..

[7] Ronnett BM, Shmooker BPM, Diennerwest M (1997). Immunohistochemical evidence supporting the appendiceal origin of pseudomyxoma in women. Int J Gynecol Pathol..

[8] Hwang JH, So KA, Modi G, Lee JK, Lee NW, Lee KW, Kim I (2009). Borderline-like mucinous tumor arising in mature cystic teratoma of the ovary associated with pseudomyxoma peritonei. Int J Gynecol Pathol..

[9] Lee KR, Scully RE (2000). Mucinous tumors of the ovary: a clinicopathologic study of 196 borderline tumors (of intestinal type) and carcinomas, including an evaluation of 11 cases with ‘pseudomyxoma peritonei’. Am J Surg Pathol..

[10] Ronnett BM, Seidman JD (2003). Mucinous tumors arising in ovarian mature cystic teratomas: relationship to the clinical syndrome of pseudomyxoma peritonei. Am J Surg Pathol..

